# Autofluorescence−spectral imaging for rapid and invasive characterization of soybean for pre-germination anaerobic stress tolerance

**DOI:** 10.3389/fpls.2024.1334909

**Published:** 2024-02-27

**Authors:** Ambika Rajendran, Ayyagari Ramlal, Subham Sarkar, Sarit S. Agasti, K. Rajarajan, S. K. Lal, Dhandapani Raju, Sreeramanan Subramaniam

**Affiliations:** ^1^ Division of Genetics, Indian Council of Agricultural Research (ICAR)-Indian Agricultural Research Institute (IARI), New Delhi, India; ^2^ School of Biological Sciences, Universiti Sains Malaysia (USM), Georgetown, Penang, Malaysia; ^3^ New Chemistry Unit, Jawaharlal Nehru Centre for Advanced Scientific Research (JNCASR), Bengaluru, Karnataka, India; ^4^ Tree Improvement Research Division, Central Agroforestry Research Institute (ICAR-CAFRI), Jhansi, Uttar Pradesh, India; ^5^ Division of Plant Physiology, Indian Council of Agricultural Research (ICAR)-Indian Agricultural Research Institute, New Delhi, India; ^6^ Chemical Centre Biology (CCB), Universiti Sains Malaysia (USM), Georgetown, Penang, Malaysia; ^7^ Department of Biology, Faculty of Science and Technology, Universitas Airlangga, Surabaya, Indonesia

**Keywords:** autofluorescence, spectral markers, soybean, susceptible, tolerant, imaging

## Abstract

The autofluorescence-spectral imaging (ASI) technique is based on the light-emitting ability of natural fluorophores. Soybean genotypes showing contrasting tolerance to pre-germination anaerobic stress can be characterized using the photon absorption and fluorescence emission of natural fluorophores occurring in seed coats. In this study, tolerant seeds were efficiently distinguished from susceptible genotypes at 405 nm and 638 nm excitation wavelengths. ASI approach can be employed as a new marker for the detection of photon-emitting compounds in the tolerant and susceptible soybean seed coats. Furthermore, the accuracy of rapid characterization of genotypes using this technique can provide novel insights into soybean breeding.

## Graphical Abstract

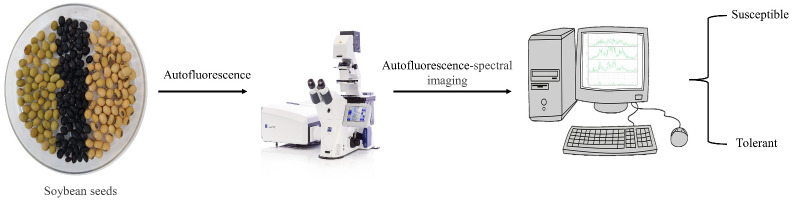

## Introduction

Stress namely salinity, heat, drought and several others including waterlogging (WL) hinder plants from achieving their full potential causing huge crop losses globally. WL causes very often anoxia, hypoxia and may affect soil fertility which at times may result in yield reduction (Pan et al., 2021; Parrotta et al., 2023; Ramlal et al., 2023a). The majority of agriculturally important crops at some point during their growth and developmental stages are prone to water stress. Arable land (~ 12% of the total) is often affected by WL resulting in 20% yield reductions which would be escalated as a result of climate change (Tian et al., 2021). Soybean is a versatile, agriculturally and industrially important legume. WL stress is critical in soybean during various stages of its development (grain-filling, emergence and germination) (Ploschuk et al., 2022; Rajendran et al., 2022, 2023; Ramlal et al., 2023a, 2023b, 2023c, 2024). Therefore, there is a crucial need for the development of novel strategies enabling the identification of superior genotypes (tolerant/susceptible) that will be helpful for sustainable crop production under different and harsh climatic conditions.

Fluorescence-based techniques have far-reaching significance in different fields of research. Traditional fluorescence imaging relies on the fluorescence of extrinsic fluorophores conjugated with the target site. On the other hand, non-fluorescent compounds can be quantified by using multiple identifiers such as fluorescent tracers, proteins or stains that can highlight the molecular, anatomical or physiological features. The latter technique assesses only a small part of a target sample otherwise called “spot measurement” so it hardly provides spatial information important for certain applications (Buschmann et al., 2000; Li et al., 2019; Donaldson, 2020; Barboza da Silva et al., 2021). Therefore, the advanced ASI method can be a better alternative tool to overcome such limitations.

ASI can be an optical tool combining spectral and spatial information as a biochemical constituent in a single detection protocol. It provides a high-resolution optical spectral peak and image at a specific excitation-emission wavelength determined by the natural fluorophore. Autofluorescent compounds also known as natural fluorophores are natural pigments (chlorophylls) and structural components (lignin) of cell walls that can be used for visualization. Once fluorescent compounds are stimulated by any source of light, they are raised to an excited state due to photon absorption and the light re-emitted is measured by the sensors wherein the photon absorption and fluorescence emission occur simultaneously. Hence, Integrating ASI technology with chemometric approaches may allow better characterization of genotypes in a non-destructive way which includes leaves, detecting seed viability and quality (Buschmann et al., 2000; Li et al., 2019; Donaldson, 2020; Galletti et al., 2020; Barboza da Silva et al., 2021). This study can widen the spatial and functional identification of compounds specific for abiotic stress in the seed coat. In the study, the use of ASI for the classification of soybean genotypes based on differences in their seed coat chemistry was examined.

## Material and methods

### Plant material

Harvested and manually threshed seeds of ten soybean genotypes used in this study were procured from the Soybean Unit, Division of Genetics, ICAR-Indian Agricultural Research Institute, Pusa Campus, New Delhi, India. Genotypes chosen for this study were already characterized as contrasting donors for waterlogging and through a series of initial experiments in the lab and land in the Division of Genetics, IARI (Rajendran et al., 2023). The study was conducted in the New Chemistry Unit of JNCASR, Bengaluru. Seed coats were detached with the help of forceps using a gentle knock to seeds placed in between muslin cloth from two replications of 20 seeds.

### Biochemical analysis of the seed coat

Being significant in waterlogging tolerance and autofluorescence, the total chlorophyll, phenol and lignin proportion present in seed coat samples of contrasting genotypes was determined. For total phenol estimation, 100 mg of seeds was mixed with 2.0 ml of 70% ethanol for 30 minutes and kept at 70°C in a water bath. Followed by centrifugation for 5 minutes at 13,000 rpm, the supernatant was collected and Folin–Ciocalteau reagent and Na_2_CO_3_ were added. The absorbance was measured at 725 nm (Swain and Hillis, 1959). Lignin in the seed coat was determined using 10 mg of powdered seed mixed with 1 ml water (98°C) for 30 minutes. The samples were centrifuged at 14,000 rpm for 5 minutes and then the supernatant was mixed with 1 ml ethanol and incubated for 30 minutes at 76°C. Additionally, 1 ml of chloroform was added to samples and incubated at 59°C for 30 minutes. At last, 1 ml of acetone was added and the samples were again incubated at 54°C for 30 minutes followed by centrifugation for 5 minutes at 14,000 rpm, The supernatant was further separated and the pellet obtained was dried for 45 minutes using SpeedVac vacuum. The purified extract was used for lignin estimation using acetyl bromide (Fukushima and Kerley, 2011). A sample of 2.5 g powdered seed coat was weighed and squashed with a small amount of 80% acetone in a dark room for chlorophyll extraction following the procedure of Pandey et al. (1989). The extract was filtered into an amber flask, washed until 15 ml of extract in an ice bath and read in a spectrophotometer at 645 and 663 nm wavelengths.

### Autofluorescence spectral imaging

To verify the presence of autofluorescence by natural fluorophores present on the seed coat, autofluorescence images for the samples were generated. Multispectral fluorescence images were captured from seed coats of all genotypes using a confocal microscope (Leica TCS SP8) with laser imaging. This system is integrated with a CCD-chip, providing autofluorescence-spectral images with the following parameters 1024 × 1024 pixels; image size: 116.25 μm × 116.25 μm; pixel size: 113.64 nm × 113.64 nm in a few seconds and requires no preparation of samples. It is equipped with five solid-state diode lasers with 405 nm, 488 nm, 552 nm and 638 nm excitation wavelengths showing emission ranges of 415-497 nm (UV light), 498-583nm (blue light), 550–599 nm (yellow light) and 647–725 nm (red) respectively. At a specific wavelength, a number of intrinsic and extrinsic fluorophores can get activated to emit light. In the study, sequential scanning was used to obtain autofluorescence spectral data using a hybrid detector. The ImageJ (FIJI) software was then used to analyze the data, removing the influence of outliers (lowest and highest 10%) of the data.

### Statistical analyses

Analyses of variance (ANOVA) were done and the least significant differences (l.s.d) at P > 0.05 were calculated for significant differences using the OPStat (Sheoran et al., 1998). Pearson’s Correlation coefficient was used to measure the relationship between different excitation-emission combinations and the seed traits using R software (Version 4.3.2) (R Core Team, 2023).

## Results

**Figure 1:**
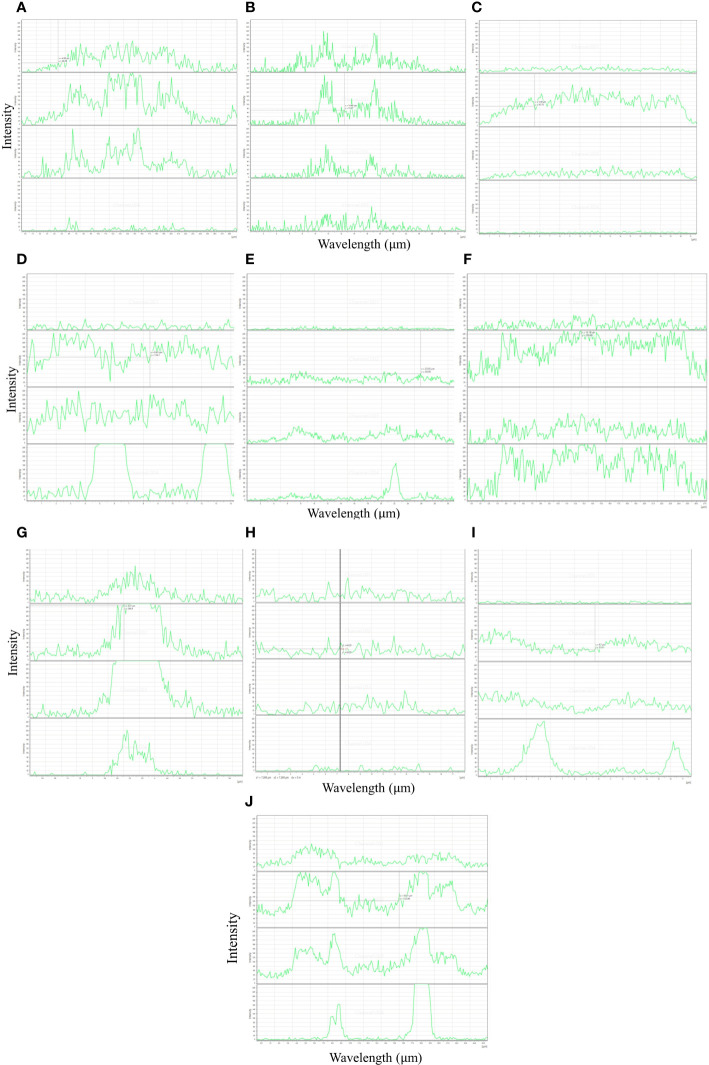
Autofluorescence intensity profile in genotype tolerant **(A–C)**, susceptible **(D–F)**, moderately tolerant **(G–J)** seed coats from soybean at four channels (405, 488, 552 and 638 nm) (Bar = 200 µm). (X axis, emission intensity; Y axis, line of projection in µm).

In the study, autofluorescence-spectral imaging of intrinsic natural fluorophores like lignin, phenols and chlorophyll was done to efficiently identify the difference of soybean genotypes to tolerate waterlogging at the pre-germination stage. The imaging was supplemented with biochemical analysis of seed coat constituents in contrasting genotypes. Tolerant genotypes EC471972 (7.9%), EC471920 (7.4%), and EC472119 (6.4%) had significantly higher amounts of lignin, whereas genotype which is highly susceptible to waterlogging at the pre-germination stage, had a significantly lower amount of lignin (**Table 1**). The distinct autofluorescent compounds present in susceptible and tolerant genotypes showed emission spectra at wavelengths 405 nm and 638 nm respectively enabling a clearcut separation of tolerance level of genotypes used (**Figure 1**).

**Table 1:** Variation in proportion of phenol, chlorophyll and lignin content of seed coat in soybean contrasting genotypes.

Genotypes	Phenols (mgg^-1^ DW)	Chlorophyll (mg/100 g DW)	Lignin (% of cell wall)
EC456616	0.7d	5.00g	2.320f
EC472119*	1.36b	18.24cd	7.900a
EC391181	0.4e	15.17ef	5.180c
EC389116**	0.05f	26.02b	1.490g
EC471805	0.04f	19.44c	5.390c
EC458369**	0.03f	27.22b	3.260e
EC471275**	0.02f	29.34a	2.280f
EC471972*	1.1c	15.12ef	7.450a
EC471920*	1.7a	16.07e	6.410b
EC471900	0.7d	13.32f	4.300d

Data represent the mean ± SD (n = 3). Significant difference among tolerant, moderate and susceptible genotypes were analyzed by ANOVA with Duncan’s multiple range test (DMRT) (P >0.05), *Tolerant and **Susceptible genotypes.

Chlorophyll autofluorescence allows rapid screening of genetic and environmental variables in large numbers of plants (Donaldson, 2020). Black-seeded varieties (EC471972, EC471920 and EC472119) tolerant genotypes (15.12 mg/100 g DW) had the lowest amount of chlorophyll in the seed coat. A significantly higher amount of seed coat chlorophyll (29.37 mg/100 g dry weight) was found in susceptible genotypes. Yellow-seeded genotypes (EC389116, EC458369, EC471275) which are most susceptible to waterlogging stress during the pre-germination stage recorded the lowest total phenol (0.02 mg*/*g). The tolerant black-seeded ‘EC471920’ (1.7 mg*/*g) had significantly higher phenol content in the seed coat than the susceptible and moderately tolerant ones. Phenol is an autofluorescent compound with an excitation peak at 273 nm and an emission peak at 300 nm. Pearson’s correlation analysis showed a negative correlation between phenols-chlorophyll and lignin-chlorophyll while a positive correlation with lignin-phenols at the excitation wavelength of 405 nm, 488 nm and 638 nm for lignin, phenols and chlorophyll respectively (**Supplementary Figures 1A, B**).

## Discussion

Lignin, a phenylpropanoid is a dominant fluorophore associated with seed coat properties (Alvarez et al., 1997). Lignin has a wide excitation range resulting in emission in the blue and green range. It can also show wide excitation at 405/488 nm and emission at 500–550 nm. Lignin is also found to be brighter than other blue fluorophores (Donaldson and Williams, 2018). Water impervious soybean seeds have a higher percentage of seed coat lignin content than absorbent ones indicating resistance to mechanical damage (Tavares et al., 1987). The primary emission of chlorophyll at 685 and 720–730 nm causes chlorophyll autofluorescence (Donaldson, 2020). The two most important autofluorescent molecules found in plants are chlorophyll and lignin however a wide range of other molecules present in cytoplasm and cell walls are also autofluorescent. Therefore, fluorescence in lignin at 405 nm and chlorophyll at 633 nm appeared to be an important indicator of soybean seed quality for the agricultural industry. Furthermore, intrinsic and extrinsic fluorophores significant in waterlogging stress like cellulose, and catalase, can be used for understanding seed coat chemistry in relation to tolerance. Emission intensity was a peak in the 488 nm excitation channel invariably in all classes of tolerance of genotypes. The green fluorescence (510–520 nm) excited by 488 nm appears to be linked with flavonoids, while yellow lighting at the excitation in region 450–490 nm seems to be linked with carotenoids and some anthocyanins (Roshchina, 2008). Understanding the spectra of fluorophores depends on choosing the right light (e.g. LED, arc lamps, laser lines) source for excitation and the right filters and detectors for emission.

## Conclusion and future prospects

The extent of seed coat biochemical compounds that can be detected by autofluorescence spectral imaging, can be used as a new marker for qualitative analysis of soybean seeds. In seed context, this approach can improve the accuracy of rapid quality tests, preliminary screening and characterization providing novel insights into soybean abiotic stress breeding. The approach of implementing autofluorescence spectral imaging in the determination and evaluation of tolerant and susceptible waterlogging genotypes in soybeans has been achieved and can be used as a new marker for qualitative analysis of soybean seeds. Moreover, this methodology may improve the functional efficacy of rapid invasive and the characterization of genotypes can be extended to other crops as well.

## Data availability statement

The original contributions presented in the study are included in the article/**Supplementary Material**. Further inquiries can be directed to the corresponding author.

## Author contributions

AmR: Conceptualization, Data curation, Formal analysis, Funding acquisition, Investigation, Methodology, Project administration, Resources, Software, Visualization, Writing – original draft, Writing – review & editing. AyR: Resources, Software, Writing – original draft, Writing – review & editing. SuS: Resources, Software, Writing – review & editing. SA: Conceptualization, Project administration, Resources, Software, Supervision, Writing – review & editing. KR: Resources, Writing – review & editing. SL: Conceptualization, Resources, Supervision, Writing – review & editing. DR: Resources, Writing – review & editing. SrS: Resources, Writing – review & editing.

## Supplementary material

Supplementary Figure 1
**(A)**. Pearson’s correlation coefficients among the genotypes, phenols, chlorophyll and lignin between all bandwidths (488nm, 638nm and 405nm) used for autofluorescence-spectral imaging of soybean seeds (n = 20 seeds). Asterisks indicate significant differences according to Duncan’s multiple range test (DMRT) at p < 0.05 (*). **(B)**. Correlation among the phenols, chlorophyll and lignin at 488nm, 638nm and 405nm respectively. Asterisks indicate significant differences according to Duncan’s multiple range test (DMRT) at p < 0.05 (*), 0.01 (**) and 0.001 (***) and non significant at p ≥ 0.05.
