# Supplementary figures and images for: Autofluorescence−spectral imaging for rapid and invasive characterization of soybean for pre-germination anaerobic stress tolerance
Source: Front Plant Sci. 2024 Feb 27;15:1334909. doi: 10.3389/fpls.2024.1334909 (PMC10927947; doi:10.3389/fpls.2024.1334909)

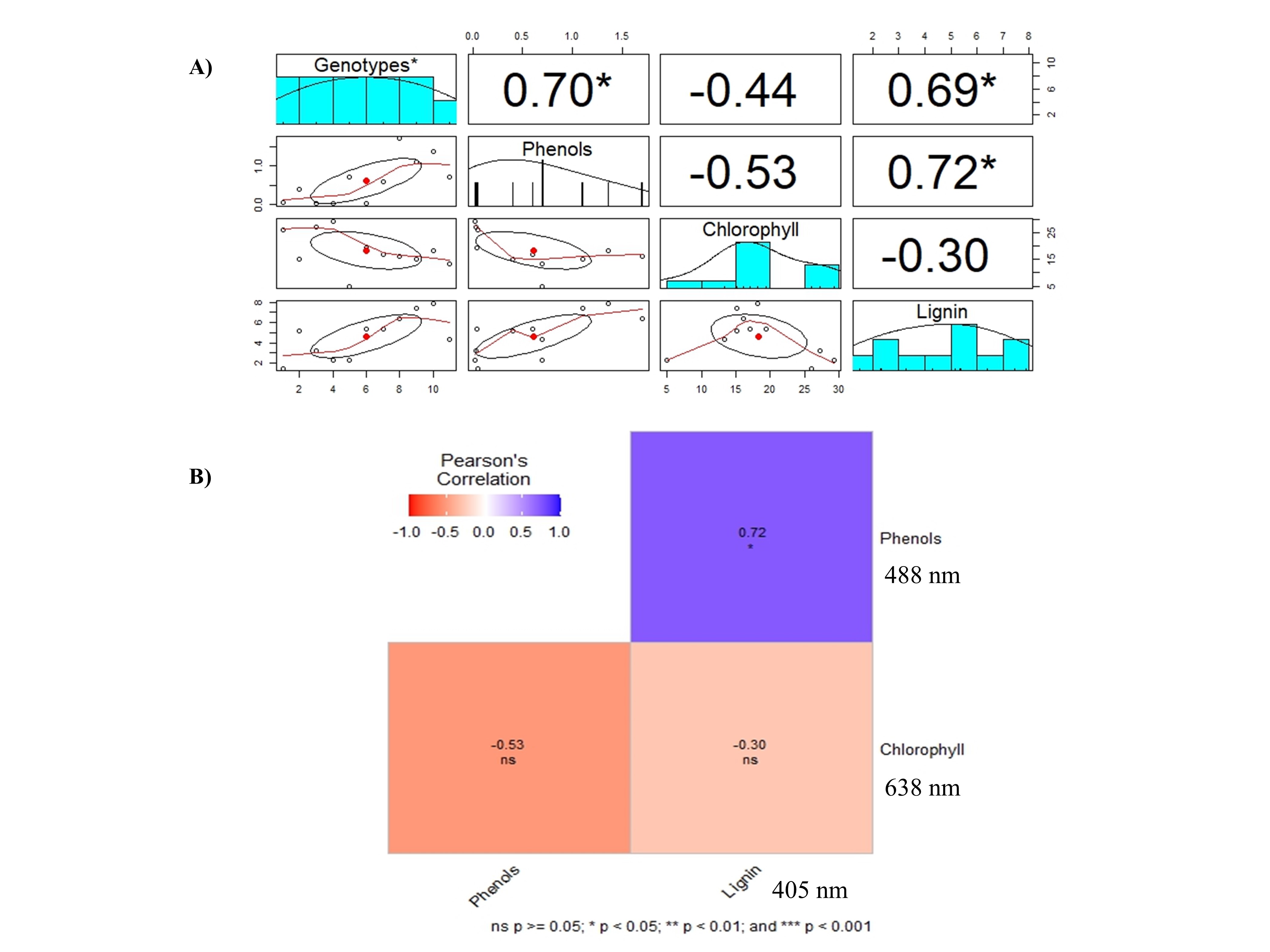

Supplement: Supplementary Figure 1 — (A). Pearson’s correlation coefficients among the genotypes, phenols, chlorophyll and lignin between all bandwidths (488nm, 638nm and 405nm) used for autofluorescence-spectral imaging of soybean seeds (n = 20 seeds). Asterisks indicate significant differences according to Duncan’s multiple range test (DMRT) at p < 0.05 (*). (B). Correlation among the phenols, chlorophyll and lignin at 488nm, 638nm and 405nm respectively. Asterisks indicate significant differences according to Duncan’s multiple range test (DMRT) at p < 0.05 (*), 0.01 (**) and 0.001 (***) and non significant at p ≥ 0.05. [file Image_1.jpeg]
